# Challenges in Translating from Bench to Bed-Side: Pro-Angiogenic Peptides for Ischemia Treatment

**DOI:** 10.3390/molecules24071219

**Published:** 2019-03-28

**Authors:** Karel Petrak, Ravi Vissapragada, Siyu Shi, Zain Siddiqui, Ka Kyung Kim, Biplab Sarkar, Vivek A. Kumar

**Affiliations:** 1 NangioTx Inc., Newark, NJ 07102, USA; petrak@nangiotx.com; 2 Department of Gastrointestinal Surgery, Flinders Medical Centre, 5042 Bedford Park, South Australia, Australia; ravi.vissapragada@gmail.com; 3 Department of Medicine Stanford School of Medicine, Stanford, CA 94305, USA; syshi@stanford.edu; 4 Department of Biomedical Engineering, Newark, NJ 07102, USA; zs67@njit.edu (Z.S.); kk564@njit.edu (K.K.K.); bsarkar@njit.edu (B.S.); 5 Department of Chemical and Materials Engineering, New Jersey Institute of Technology, Newark, NJ 07102, USA; 6 Rutgers School of Dental Medicine, Newark, NJ 07103, USA

**Keywords:** angiogenesis, peptide, translation, ischemia, therapeutic, development

## Abstract

We describe progress and obstacles in the development of novel peptide-hydrogel therapeutics for unmet medical needs in ischemia treatment, focusing on the development and translation of therapies specifically in peripheral artery disease (PAD). Ischemia is a potentially life-threatening complication in PAD, which affects a significant percentage of the elderly population. While studies on inducing angiogenesis to treat PAD were started two decades ago, early results from animal models as well as clinical trials have not yet been translated into clinical practice. We examine some of the challenges encountered during such translation. We further note the need for sustained angiogenic effect involving whole growth factor, gene therapy and synthetic growth factor strategies. Finally, we discuss the need for tissue depots for de novo formation of microvasculature. These scaffolds can act as templates for neovasculature development to improve circulation and healing at the preferred anatomical location.

## 1. Introduction

Ischemia is a serious complication of peripheral artery disease (PAD). This disease is caused by atherosclerosis, which begins with endothelial injury leading to deposition of lipids and calcium, and the consequent stenosis of the vessel lumen [1,2]. Atherosclerosis can occur in any blood vessel, however, severe tissue damage from atherosclerosis is usually due to occlusion of vessels that have few to no collaterals, or those that supply organs with high demand. For example, commonly in PAD, stenosis of the femoral artery (or its branches) severely reduces flow to the lower limb, resulting in symptoms ranging from mild claudication during exertion, to limb-threatening ischemia, even tissue necrosis warranting consequent amputation [2]. Ischemia induces both local and systemic responses. Understanding the theoretical principles governing the underlying mechanism and physiologic self-repair has led the current and potential therapeutic applications.

The prevalence of PAD is between 3 and 10% in the general population, and up to 15–20% in the elderly [2,3]. Pharmacologic treatments have had limited success when the disease has advanced to critical limb ischemia (CLI). Currently, management of PAD is predominantly based on lifestyle modification, specifically exercise and smoking cessation [4,5]. This reduces the causative elements that induce atherosclerosis. Pharmacologic options are limited and mainly relate to risk factor reduction, such as lowering blood pressure and concentration of low-density lipoprotein cholesterol (LDL-cholesterol) and glucose in blood. Anti-platelet therapy can offer some benefit reducing acute occlusion of microvasculature [6]. Surgical approaches include endovascular angioplasty and stenting or endarterectomy to reduce narrowing. Synthetic and vein graft procedures carry inherent risks, especially synthetic grafts and below-knee implants [7]. Ultimately, life-saving limb amputation may be the only option available in certain patients who do not qualify or are refractory to the above interventions.

## 2. Strategies for Therapeutic Vascularization

### 2.1. Angiogenic Therapies

Claudication symptomatic to occlusive vessel disease can also potentially benefit from improved microvascular perfusion. While certainly not addressing large vessel disease, microvascular disease management can lead to improved outcome for patients with intermittent claudication, early stage limb ischemia, and impaired wound healing. This provides a safe method of revascularization that ensures stable, viable and durable vasculature to support limb microvasculature already damaged by PAD. In regard to ischemia, many local and systemic responses have been studied (Table 1). Three main angiogenic growth factors responsible for alleviating ischemia have been utilized in randomized controlled trials, mostly compared to placebos or standard treatment: vascular endothelial growth factors (VEGF), fibroblast growth factor (FGF) and hepatocyte growth factor (HGF). There are other growth factors included in several trials, such as platelet-derived growth factor (PDGF) and placental growth factor (PLGF). Our research focused primarily on VEGF, as it is one of the best-characterized angiogenic growth factors [8,9,10,11,12,13]. Out of the various VEGF isoforms that differ by their amino acid length, VEGF-A121 and VEGF-A165 are most widely studied in clinical trials [14].

The first case of therapeutic angiogenesis in a patient with PAD was reported in 1996 by Isner et al. [8]. Several phase 1 studies reported promising results and enabled subsequent phase 2 studies. Mohler et al. developed CI-1023 (AD_GV_VEGF121.10), a recombinant adenovirus vector belonging to the adenovirus 5 genome family [15]. They conducted a phase I trial to test the efficacy of CI-1023 when injected intramuscularly, and study population were patients with PAD over the age of 35 years. Patients were injected intramuscularly 20 times with 1 mL of CI-1023 to the area of ischemia in the lower limb. At the conclusion of this study, they noted that the CI-1023 was safe to use as an angiogenic therapy, however, due to patient drop-out and other complications, the efficacy of their therapy could not be accessed and that larger randomized clinical trials were required to gauge efficacy.

Other studies using FGF and HGF have shown significance such as Nikol et al.’s plasmid-based gene delivery system, NV1FGF, based on fibroblast growth factor 1 (FGF-1) [16]. In a phase 2 trial, NV1FGF was administered intramuscularly in a randomized, double-blind, and multinational study consisting of 125 patients suffering from CLI. The patients involved in this study could not be considered for revascularization as a viable option partly due to non-healing ulcers. The primary endpoint of this study was the total healing of one ulcer by week 25 and the secondary end points included a lower ankle-brachial index, amputation, and death. These patients were injected intramuscularly eight times with either NV1FGF or a placebo at day 1, 15, 30, and 45. At the conclusion of this study, the ulcer healing rates were similar for both NV1FGF (19.6%) and the placebo (14.3%). However, NV1FGF reduced the risk of amputation two-fold compared to the placebo. Even though the primary endpoint was not met, Nikol et al. [16] stated that they will continue to a phase 3 study with amputation as the primary endpoint. A summary of VEGF and angiogenic peptide related studies has been presented in Table 1. There have been approximately 20 trials with these factors. We have chosen to present the key studies to highlight the differences.

### 2.2. Challenges and Limitations

Therapeutic angiogenesis has been recognized as a promising therapy for patients with PAD [27,28,29]. Understanding the past efforts and data with developing angiogenesis-active entities, their formulation and delivery, is critical for the future success of angiogenesis-focused translational programs.

### 2.3. Design from a Pharmacokinetic Perspective

Pharmacokinetics, often times referred to as how the body reacts to a drug, plays a crucial role in the overall effectiveness of any drug therapy [30]. Drugs administered orally or systemically reach the disease target (limb microvasculature in this case) through biodistribution. Typically, only a fraction of the dose reaches the disease target, and the drug is removed from the body according to its pharmacokinetic half-time. Frequently, studies have elected to use intramuscular injections, specifically at sites surrounding stenoses [28], in the hopes of using the lower interstitial flow in the muscle as a drug reservoir (compared to subcutaneous injection). Injected drugs or biologic factors at the site of pathology may disperse rapidly away from the site, reducing its concentrations soon after the drug is administered. Even with optimal route and site of administration, various other factors could influence the effectiveness of angiogenic compounds. The pharmacokinetics of the preparation is critical in determining therapeutic activity of all drugs, and in the case of synthetic VEGF, its rapid biodistribution and a relatively short half-life in circulation leads to a rapid escape of free VEGF away from its site of administration, resulting in poor local efficacy and possibly systemic side effects [31]. This also applies to generating VEGF by gene transfer which, in addition of being hindered by the pharmacokinetics of free VEGF, is further limited by low levels of gene expression, induced inflammation responses to the formulation/carrier, and a lack of adequate control over the level and duration of expression [31].

### 2.4. A Dearth of Translatable Animal Models

In developing angiogenic therapeutics, a major hindrance is the inadequacy of representative in vivo models. While larger animal models do exist, none truly recapitulate lower-limb ischemia and microvascular damage in humans. In addition, obliteration of large feeder vessels to induce a PAD phenotype has generally been an ineffective model. Rabbit hind limb ischemia models have been used with injections of previously mentioned angiogenic factors. Some studies have had success in measured outcomes such as capillary density on immunohistochemistry, calf blood pressures, and laser Doppler measurement of blood flow [32,33]. However, other studies have not reported such optimistic results [34,35]. Consequently, investor tepidity in the PAD space (where devices are often tested in higher porcine, ovine, or non-human primate) have also contributed to delayed preclinical investment and development. Further, there is an obvious disparity between the biology of animal models and that of patients selected for trials of therapeutic angiogenesis [4]. An assessment of the currently available pre-clinical animal models shows major limitations resulting from variability in the surgical procedure used to induce limb ischemia, variability in the strains of rodents used, and the absence of standardized functional outcomes. Most current pre-clinical models are designed to produce acute ischemia, which leads to major muscle necrosis and acute inflammation. On the other hand, patients typically present with chronic ischemia, which has entirely different pathophysiology and associated compensatory local and systemic responses. The effects of comorbidities on these compensatory mechanisms are also not recapitulated well in these models. Better representative models, albeit challenging, may be necessary for translating potential therapeutic modalities into clinical practice. Peripheral artery disease patients are often elderly patients who have exhausted standard therapeutic options including revascularization surgeries. It is reasonable to surmise these patients differ in producing natural angiogenic responses and are less likely to respond to angiogenesis-based therapy. The selection of efficacy endpoints will certainly be very different when comparing animal experiments with testing in humans. VEGF’s function in angiogenesis is not completely understood and may cause complications. Ozawa et al. [29] found that varying the dosage of VEGF can induce either normal angiogenesis or aberrant vessels/hemangiomas. Further, they found that the local level of VEGF in the tissue microenvironment and not the overall delivered dose was critical to neovascularization; the dosage of VEGF transferred to the diseased tissue must be carefully controlled to not only provide therapeutic benefit but also avoid off-site complications.

### 2.5. RNA Based Techniques

Bonauer et al. utilized microRNAs (miR), which are a class of noncoding RNA that serve a key function in the regulation of gene expression, to study their effects on enhancing blood vessel growth in a murine ischemia model [36]. microRNAs bind to specific messenger RNA (mRNA) to induce mRNA degradation, enabling researchers to adjust physiologic actions. Bonauer et al. [36] conducted a miR profiling study and found that the miR-17~92 cluster is expressed in human endothelial cells. Overexpression in one subset of this cluster, miR-92a, inhibited sprout formation in spheroids of human endothelial cells and the hemoglobin count reduced considerably in an in vivo matrigel plug study in mice. Their group then tested whether the inhibition of miR-92a could potentially promote neovascularization. They designed antagomir-92a to prevent the binding of miR-92a. They first determined whether the expression levels of miR-92a increased due to ischemic injury in a mouse hind limb ischemia model; they found levels were considerably higher, especially within the first 3 days post injury. Injection of antagomir-92a induced a significant reduction in toe necrosis compared to the phosphate-buffered saline (PBS) and antagomir controls. In addition, blood flow recovery and the amount of smooth muscle actin-positive arterioles and capillaries were enhanced in the antagomir-92a treated mice. This experiment demonstrated that antagomir-92a facilitated perfusion to the ischemic limbs in those mice. However, the inhibition of miR-92a in their mice ischemia model required five injections over a 9-day period. A therapeutic option that offers a consistent long-term release may be more convenient.

### 2.6. Mimicking Nature’s Angiogenic Scaffolds

A number of strategies have been investigated for the presentation of VEGF signaling in scaffolds; self-assembling peptide hydrogels presenting high density of epitopes mimicking VEGF and other growth factors have been developed [37,38,39,40,41,42,43,44,45,46,47,48,49,50,51,52,53,54,55,56,57,58,59,60,61]. Using mimics of VEGF, large macromolecules can be functionalized. These mimics have been designed for inducing angiogenesis and treating poor blood flow in ischemic muscles. The peptide backbone self-assembles and forms hydrogel in situ upon injection of peptide solution into tissue. An example of a self-assembling peptide bearing VEGF mimetic sequences that forms such a hydrogel is the drug SLanc. This material consists of a novel multi-domain peptide sequence of 31 amino acids, with each of the domains serving a specific purpose [62,63]. The peptide is easily produced by solid-phase peptide synthesis, and its shelf life at room temperature is longer than a year in a standard laboratory environment. An important feature of the peptide design is that SLanc self-assembles into a nano-fibrous matrix that is compatible with the natural extracellular matrix. This peptide matrix provides a durable and protective courier for delivering peptides, which even when sheared can readily reform due to the nature of the hydrophobic interactions and hydrogen bonds between individual peptide molecules. Domains of the SLanc peptide have been designed to enable peptide-controlled degradation, cell adhesion, and local stimulation of angiogenesis. Given these favorable material properties, SLanc and other synthetic growth factors remain at the site of injection for three weeks and imparts a sustained therapeutic effect. This stable hydrogel provides a scaffold for the VEGF receptors and resists rapid dispersion. Enzymatic breakdown is also reduced with the help of the hydrogel extracellular matrix [40,45]. These matrices circumvent many of the challenges expressed earlier by maintaining and presenting VEGF at a fixed pre-determined anatomical location long enough to ensure development of new functional microvasculature [37,62]. This attracts cells that are crucial for the de novo formation of microvasculature to the treated anatomical location. These properties of such hydrogels address major limitations with current plasmid and viral delivery mechanisms, which we believe are fundamental to improving flow to ischemic areas [64].

### 2.7. Safety Prior to Clinical Evaluation

Safety considerations are of utmost importance especially when translating technologies towards humans [65]. There are potential dangers of pathological angiogenesis in non-target tissues from diffusion of non-depot confined angiogenic factors. This has led to resistance (and often exclusion criteria in clinical trials) of the implications of VEGF in the pathogenesis of ocular conditions such as proliferative diabetic retinopathy, ischemic central retinal vein occlusion, retinopathy of prematurity, and oxidative, age-related macular degeneration. Concerns about the possible harm of pro-angiogenic factors on atherosclerosis and malignancies were not confirmed by human in vivo data. However, it is relevant to note that patients with a history of cancer or active malignancy are uniquely excluded from most PAD trials (www.clinicaltrials.gov).

### 2.8. Localizing a Signal

Conventional drug-delivery depots developed for local administration of small molecules may release free VEGF into the bloodstream and affect distant organs. Self-assembling peptide scaffolds designed as drug-delivery systems can present a chemically immobilized functional mimic of a growth factor on a hydrogel base, such as SLanc above, that can attract key cells responsible for angiogenesis. This signaling is essential for cell survival and biological function [66]. This unique approach to treating ischemia is based on molecular mimicry of the body’s natural mechanism for blood-vessel generation. The process is mediated by the engagement of the VEGF to its cognate receptors. Self-assembling peptides have shown efficacy in preclinical hind limb ischemia models [40], with improvement of blood flow in limbs in 8-month and 13-month old mice seen in just seven days. In phase 2 clinical trials for PAD, improvement in Doppler imaged perfusion and treadmill endurance is measured. [40].

### 2.9. Influence of Stakeholder Understanding on Drug Development

The process of translating research results into development of new therapeutic products is fraught with many obstacles. Some stem from the fact that the process of discovery in academia is focused on innovation and is not concerned with data validation and practicality of newly created ideas. Another major hindrance is the difficulty of securing the necessary resources to embark on a product development. It is of paramount importance for a research-based innovative start-ups to fully understand what has been done in the selected indication to date, especially which failures occurred and why, establish a clear case for how progress could be made in future, and find ways of explaining this to the business community in terms it understands. A thorough consideration of scientific and market risks involved and systematic step-wise removal of these risks during preclinical development through generation, formulation, and testing of underlying hypotheses should be emphasized. With so much promise and therapeutic benefit at stake, it is crucial to bridge the gap between the scientific and business community to translate academic research into bedside therapies.

## 3. Conclusions

Ischemia consequent to PAD or CLI is primarily caused by atherosclerotic plaques that cause inflammation in the walls of blood vessels. Large vessel disease is managed well with angioplasty, stenting, endarterectomy, or bypass grafting. Microvascular disease deep in tissue beds is only symptomatically managed pharmacologically. Peripheral ischemic vasculopathy requires neovascularization strategies to decrease ischemic burden. Although pharmaceutical and academic researchers have worked on development of pro-angiogenic treatments, none have been brought successfully to the market. Growth factors such as VEGF, FGF, and HGF are the most commonly utilized angiogenic growth factors in angiogenic therapy trials. A number of gene therapy and RNA therapies have also been investigated. Due to diffusion from the target site, only a small fraction of the therapeutic dose reaches the target site if systemically administered, while drugs injected at the site may have adverse systemic side effects due to rapid diffusion from tissue depots.

With respect to clinical translation of technologies, a lack of consistent results in vector-based angiogenic treatments of large animal disease models that cannot accurately demonstrate clinical manifestations of chronic ischemia (and co-related co-morbidities) has made it challenging to garner resources. Self-assembling hydrogels present an injectable multi-functional platform consisting of a self-assembling backbone and VEGF mimic domains. Localization of in situ boluses of activated hydrogel therapies may reduce frequency of injections and prevent rapid dispersion from area of treatment. On a final note, efforts at industry, private capital and research institutions must be integrated for the success of angiogenic, and more generally, any biotherapeutic strategies.

## Figures and Tables

**Table 1:** Summary of recent vascular endothelial growth factors (VEGF) and angiogenic vector-based strategies.

Author	Year	Vector	Treatment	Route	Dosing
Deev et al. [17,18]	2015	Plasmid	VEGF165	IM	4–5x Day 1 and 14
Rajagopalan et al. [19]	2003	Viral	VEGF121-Ad	IM	Low dose, high dose, single session
Mohler et al. [15]	2003	Viral	VEGF121	IM	20x
Makinen et al. [20]	2002	Both	VEGF-Ad, VEGF PL	IA	2x following PTA
Deev et al. [17,18]	2017	Plasmid	VEGF165	IM	4–5 injections
Kusumanto et al. [21]	2006	Plasmid	VEGF	IM	4x Day 0, 28
Nikol et al. [16]	2008	Plasmid	NV1FGF	IM	8x Day 1, 15, 30, 45
Belch et al. [22]	2011	Plasmid	NV1FGF	IM	4x Day 1, 15, 29, 43
Powell et al. [23,24]	2010	Plasmid	HGF	IM	8x Day 0, 14, 28
Shigematsu et al. [3]	2010	Plasmid	HGF		8x day 0, 28
Kibbe et al. [25,26]	2016	Plasmid	HGF	IM	Day 0, 14, 28, 42
Rajagopalan et al. [19]	2007	Viral	Ad2 HIF	IM	-

VEGF: Vascular Endothelial Growth Factor; IM: intramuscular; IA: intra-arterial.
